# Effect of remote ischaemic conditioning on contrast-induced nephropathy in patients undergoing elective coronary angiography (ERICCIN): rationale and study design of a randomised single-centre, double-blind placebo-controlled trial

**DOI:** 10.1007/s00392-013-0637-3

**Published:** 2013-11-29

**Authors:** Robert M. Bell, Roger Rear, John Cunningham, Anne Dawnay, Derek M. Yellon

**Affiliations:** 1 The Hatter Cardiovascular Institute, University College London, 67 Chenies Mews, London, WC1E 6HX UK; 2 Centre for Nephrology, UCL Medical School, Royal Free Campus, Rowland Hill Street, London, NW3 2PF UK; 3 Department of Biochemistry, University College London Hospitals NHS Trust, 60 Whitfield St, London, W1T 4EU UK

**Keywords:** Remote ischaemic conditioning, Preconditioning, Contrast-induced nephropathy, Acute kidney injury, Clinical trial, Coronary angiography, Percutaneous intervention

## Abstract

**Background:**

Contrast-induced nephropathy (CIN), an acute kidney injury resulting from the administration of intravascular iodinated contrast media, is an important cause of morbidity/mortality following coronary angiographic procedures in high-risk patients. Despite preventative measures intended to mitigate the risk of CIN, there remains a need for an effective intervention. Remote ischaemic conditioning (RIC), where non-injurious ischaemia is applied to an arm prior to the administration of contrast, has shown promise in attenuating CIN but its effectiveness in preserving long-term renal function is unknown, which will be studied as part of the effect of remote ischaemic conditioning against contrast-induced nephropathy (ERICCIN) trial. (http://Controlled-trials.com Identifier: ISRCTN49645414.)

**Methods:**

The ERICCIN trial is a single-centre, randomised double-blinded placebo-controlled trial which plans to recruit 362 patients who are at risk of CIN, defined by pre-existent renal impairment (estimated glomerular filtration rate <60 ml/min/1.73 m^2^), over a period of 2 years. Patients will be randomised to either control or RIC consisting of 4, 5 min 200 mmHg balloon-cuff inflation/deflations, to the upper arm. The primary endpoint will be the development of CIN (>25 % of eGFR, or rise of creatinine of >44 μmol/l) at 48 h. A key secondary endpoint will be whether RIC impacts upon persistent renal impairment over the 3-month follow-up period. Additional secondary endpoints include the measurement of serum neutrophil gelatinase-associated lipocalin and urinary albumin at 6, 48 h and 3 months following administration of contrast.

**Implications:**

Findings from ERICCIN trial will potentially demonstrate that RIC attenuates contrast-induced acute and chronic kidney injury and influence future clinical practice guidelines in at-risk patients undergoing coronary angiographic procedures.

## Background

Contrast-induced nephropathy (CIN) is an acute, iatrogenic kidney injury triggered by the intravascular administration of iodinated contrast agents. Defined as a 25 % rise or an absolute 0.5 mg/dl (44 μmol/L) increase in serum creatinine within 72 h of contrast exposure, in the absence of an alternative explanation [1], CIN is an important clinical problem following angiography and percutaneous intervention. It represents the third most common cause of hospital-acquired acute renal failure [2, 3] and is associated with significant mortality and morbidity, with up to 19 % of those suffering CIN developing persistent renal impairment months after contrast media exposure [4]. With the incidence of renal replacement therapy estimated at 0.2 % [5], the attendant healthcare costs associated with CIN are substantial [5]. Moderate to severe chronic renal impairment (estimated glomerular filtration (eGFR) <60 ml/min or a creatinine of >120 mmol/L; National Kidney Foundation chronic kidney disease (CKD) stages 3 and 4 [6]) is regarded as the most important risk factor in the development of CIN [7], but other risk factors such as diabetes, reduced left ventricular systolic function, advancing age, concomitant use of nephrotoxic drugs, large contrast agent volumes, intraprocedural hypotension and either high or low haematocrits (dehydration/anaemia) are also well recognised [8]. Due to the association between renal and cardiovascular disease, patients with CIN risk factors are common within the cohort of patients requiring an angiographic intervention and, despite the introduction of a number of prophylactic measures, the incidence of CIN remains unacceptably high. In the CARE study, the incidence of CIN despite optimal medical therapy remained between 10 and 15 % [9]. The notion of optimal medical care in this patient cohort remains hotly contested. There are a number of national and international guidelines and all recommend avoidance of nephrotoxic drugs, adequate pre-hydration and the use of low- or iso-osmolar contrast agents. The augmentation of pre-hydration with antioxidants or alkalinizers such as *N*-acetyl cysteine and sodium bicarbonate, respectively, remain hotly contested, but recent meta-analyses appear to suggest a small advantage towards bicarbonate pre-hydration over *N*-acetylcysteine [10]. Given a lack of clarity regarding the most efficacious intervention against CIN, a large, multicentre randomised control study has started recruiting (target 8,680 patients, the prevention of serious adverse events following angiography (PRESERVE) trial [11]), the results of which will hopefully help to clarify optimal preparation of patients at risk of CIN. Similarly, there is debate over which contrast media is the least injurious to the at-risk kidney. The landmark comparison trial between iso-osmolar iodixanol and the low-osmolar iohexol contrast media by Chalmers and Jackson in 1999, which demonstrated a modest superiority for iodixanol, has been a powerful influence on subsequent contrast media decision-making, although more recent meta-analyses seem to suggest parity of risk between iso-osmolar and the majority of low-osmolar contrast media [12–16]. The lack of clear evidence of one intervention over another appears to suggest one thing: the lack of a single successful intervention to prevent CIN. There is, therefore, a clear clinical need for an effective intervention that can reduce the incidence of CIN following angiographic and other iodinated contrast requiring procedures.

While the pathophysiology of contrast-induced renal injury remains the subject of on-going basic research, one of the principal mechanisms for acute contrast nephropathy is thought to be vaso-constrictive renal hypoxic injury and release of reactive oxygen species (ROS) [17]. Such an injury is likely to be mediated by cell-death signalling of the type found in many other organ systems typified by lethal reperfusion injury [18]—and as such may be amenable to interventions such as ischaemic preconditioning (see review [19]). Interestingly, the potential of remote ischaemic conditioning was highlighted in a retrospective study by Whittaker and Przyklenk [20], and more recently three prospective trials have been published to support the supposition of remote renoprotection. In the first, Er et al. demonstrated in a small (*n* = 50/group) proof-of-concept trial that remote ischaemic conditioning (RIC), consisting of 4 cycles of 5 min ischaemia/5 min reperfusion of the upper limb by inflation/deflation of a blood pressure cuff prior to contrast exposure, was effective in significantly reducing the rate of CIN from 40 % in controls to 12 % [21]. The second, Deftereos et al. recruited 225 non-ST elevation myocardial infarction (NSTEMI) patients to receive either sham or an ischaemic conditioning stimulus consisting of 4, 30 s ischaemia/30 s reperfusion cycles by inflation/deflation of an intracoronary balloon following intervention upon the culprit lesion; consistent with upper limb RIC, they demonstrated that intracoronary RIC reduced the rate of CIN from 29.5 % in control to 12.4 % [22]. The third, Igarashi et al. [23], using an identical conditioning regime to that used by Er et al. and, utilising a novel bio-marker, urinary liver-type fatty acid-binding protein (L-FABP) as a primary endpoint, demonstrated a significant reduction of CIN from 26.9 to 7.7 % in the remote ischaemic preconditioned group. Although a small trial (*n* = 20 per group), there was an attempt to uncover the mechanism of contrast media renal injury, with the demonstration of attenuated oxidative stress as measured by derivatives of reactive oxidative metabolite (D-ROM) levels and reduced asymmetrical dimethylarginine (ADMA), an endogenous inhibitor of nitric oxide synthase following remote ischaemic preconditioning. These data are encouraging, but there is a need for further evidence before routine medical practice can be altered. These previous proof-of-concept trials have been small and relatively limited in scope, concentrating primarily upon the incidence of acute kidney injury and have not looked at the longer-term sequelae of that renal injury in terms of persistent renal damage. As noted above, CIN is not a purely transient phenomenon; it frequently has long-term serious sequelae with persistent kidney damage evident in a fifth of those with an acute biochemical manifestation of CIN [4]. The effect of remote ischaemic conditioning against contrast-induced nephropathy (ERICCIN) trial is therefore designed to evaluate RIC against CIN in elective angiography/PCI, and to ascertain the longer-term efficacy in terms of prevention of persistent renal injury at 3 months determined by serum creatinine/estimate glomerular filtration rate and development of proteinuria, a known consequence of CIN [24] and a portent for increasing cardiovascular risk and all-cause mortality [25].

## Methods

### Study objectives

The primary objective of this study is to determine whether remote ischaemic conditioning attenuates the rate of CIN arising from the administration of iodinated intracoronary contrast as part of routine and semi-emergent (NSTEMI) coronary artery angiography/percutaneous intervention. The study will ascertain whether acute injury at 48 h following the administration of contrast, as determined by serum creatinine, serum neutrophil gelatinase-associated lipocalin (NGAL, determined by both laboratory and point-of-care testing) and new-onset proteinuria, is attenuated by remote ischaemic conditioning. The study will also seek to ascertain whether attenuation of contrast-induced nephropathy results in chronic kidney damage defined by persistently depressed glomerular filtration rate and increased or new-onset proteinuria.

### Study design

The study has received Ethical Committee approval (National Research Ethics Service Committee, London-Queen Square; REC reference 13/LO/0502). The ERICCIN trial is a placebo-controlled, single-centre double-blind proof-of-concept study. It investigates the effect of remote ischaemic conditioning upon acute renal injury at 48 h and as a secondary endpoint, to determine whether it impacts upon long-term renal function 3 months post-procedure.

### Study population

**Fig. 1:**
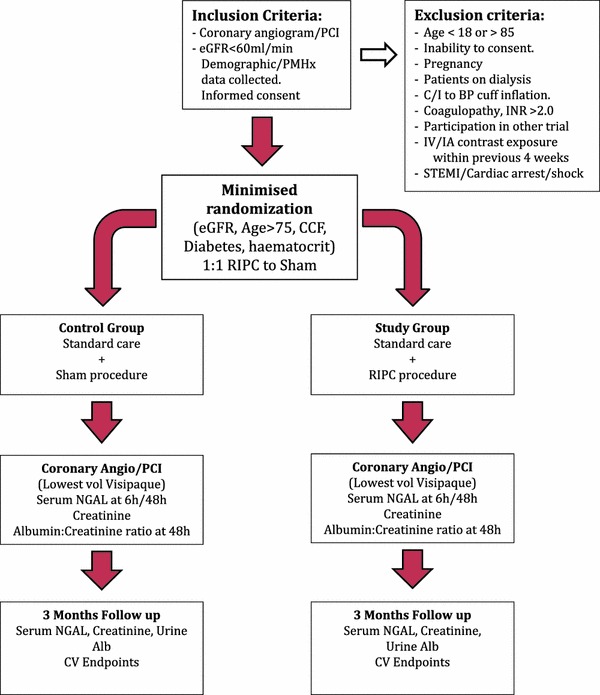
Flow diagram showing the recruitment and design of the ERICCIN trial. Patients meeting the inclusion criteria, and having provided informed consent are then randomised to either placebo (non-occlusive low-pressure inflation of the blood pressure cuff to 15 mmHg) or treatment with RIC (200 mmHg cuff-inflation). Each 5 min inflation/5 min deflation is repeated over 4 cycles. Blood samples are drawn for biochemical assessment at baseline (pre-treatment), at 6 h, at 48 h and at 3 months. At these time points, urine samples are also taken for urinary protein assessment

The investigators will seek to recruit patients aged between 18 and 85 years of age and scheduled for any Cardiac Catheter Laboratory investigation that involves the administration of intracoronary iodinated contrast at the Heart Hospital, UCLH NHS Trust (Fig. 1). The patients recruited will have an estimated glomerular filtration rate (as estimated using the modification of diet in renal disease (MDRD) formula [26]) of less than 60 ml/min/1.73 m^2^ and will have freely given informed consent. Patients excluded from the trial will fall outside these pre-requisites, and in addition will have one or more of the following exclusion criteria: pregnancy; end-stage renal failure and on dialysis; coagulopathy with an INR >2.0; recent intravenous or intra-arterial iodinated contrast exposure within the previous 4 weeks; any contraindication for balloon-cuff inflation or have presented as an ST elevation myocardial infarction, cardiac arrest or are in cardiogenic shock.

The study will conform to the spirit and letter of the declaration of Helsinki and in accordance with the UCL Good Clinical Practice Guidelines.

### Intervention

The remote ischaemic conditioning intervention will be undertaken within 2 h prior to the administration of iodinated contrast media. Those patients randomised to receive RIC will have a standard blood pressure cuff placed on the upper arm. The administration of the intervention will be via the automated delivery of four cycles of 200 mmHg blood pressure cuff inflation for 5 min followed by cuff deflation for 5 min, using a laptop-controlled device developed and purpose built within the department of Medical Physics and Bioengineering at UCL. The placebo group will receive a sham protocol similar to RIC, but rather than the cuff being inflated to 200 mmHg to induce transient limb ischaemia, the cuff is instead inflated to a non-occlusive 10 mmHg to provide the impression of a treatment being administered.

### Randomisation and allocation

On the day of the angiographic procedure, the patients will be randomised to one of two groups: remote ischaemic conditioning or control, using minimization for CIN risk factors in a 1:1 ratio. The minimization criteria are those factors which predict the risk of acute kidney injury: eGFR (<20, 20–40, 40–60 ml/min), age (<75, >75 year), diabetes, NYHA III/IV heart failure and haematocrit (<0.39, >0.39) as identified by Mehran et al. [8], and whether the patient is undergoing bi-ventricular device implantation, a recognised high-risk group for CIN [27]. This will ensure that the study and placebo groups are evenly matched in terms of the pre-procedure risk of CIN. Randomisation will be performed by a non-blinded cardiac research nurse using a freeware computer programme (MinimPy 0.3).

## Study endpoints

### Primary clinical endpoint

The primary endpoint of the trial will be the incidence of contrast-induced nephropathy, as defined biochemically by a 25 % increase or an absolute rise of 0.5 mg/dl (44 μmol/l) from baseline in serum creatinine 48 h following contrast medium exposure.

### Secondary clinical endpoints

The secondary endpoint of the study will be the demonstration of a persistent benefit of the RIC intervention against long-term renal impairment. The renal function will be determined by biochemical markers: creatinine, eGFR and serum NGAL from baseline at 6, 48 h and 3 months post-contrast exposure. These data will be further supported by the measurement urinary albumin (dipstick and Albumin:Creatinine ratio) from baseline at 48 h and 3 months post-contrast medium exposure as a measure of renal tubular function.

In addition to biochemical assessment of renal function, for all groups the cardiovascular endpoints include death, non-fatal MI, revascularisation, acute heart failure, non-fatal stroke, major haemorrhage, rehospitalisation, haemofiltration or haemodialysis during 3 months follow-up will also be recorded.

### Sample size calculation

There will be two arms to the ERICCIN trial: placebo control and remote ischaemic conditioning. We plan to recruit 362 patients (181 per arm of the study), undergoing angiographic procedures at the Heart Hospital, UCL. The rate of CIN in previous trials has ranged from 7 % [8] to 40 % [21] (Table 1), with a mean and median reported rate of CIN of 19 and 17 %, respectively. These data are in-line with our own audit data of 1,913 patients undergoing angiographic procedures at the Heart Hospital over the period of 1st June 2011 to the 30th June 2012, revealing a rate of CIN of 22 % over the study period (unpublished data). For our study calculations, we proposed a CIN incidence of 15 %.

**Table 1:** Summary of CIN incidence in published clinical trials

Study	Number of patients recruited	Incidence of contrast nephropathy in control group (%)
RenPro [21]	100	40
Renoprotective effect of RIC [22]	225	29.5
Iloprost prevents CIN [29]	208	22
Cystatin-C and CI-AKI [35]	410	21.2
Persistent renal damage after CI-AKI [4]	3,986	12.1
CARE study [9]	482	10
REMEDIAL trial [28]	326	9.9
Urinary IL-18 and NGAL as early predictive markers [36]	150	8.7

Citations are listed in order of cited CIN incidence

Two previous studies have evaluated RIPC and its effect on CIN, reporting between 58 % [22] and 70 % [21] reduction of acute kidney injury. For our power calculations, we have proposed an effect at the lower end of this range, and have powered our study to determine a difference of 60 %. For a power of 80 % and a significance level of 0.05, we would need to recruit 181 patients into each trial arm, 362 patients in total.

### Statistical analysis

The effect of remote ischaemic conditioning on CIN will be evaluated using logistic regression. This will be adjusted for the minimization factors, as well as other covariates of interest (gender, volume/type of contrast, peri-procedural hypotension and intra-arterial balloon pump use).

The effect of RIPC treatment on serum creatinine, eGFR and urine ACR compared with baseline, at 48 h and 3 months will be evaluated using a repeated measures mixed effects model. The effect on serum NGAL compared with baseline at 6, 48 h and 3 months will be evaluated using the same model. The treatment effect will be adjusted for minimization factors and other covariates of interest (gender, volume/type of contrast, peri-procedural hypotension and intra-arterial balloon pump use.) A Bonferroni correction will be applied to eGFR ranges (<20, 20–40, 40–60 ml/min). The data analysis will be performed in a blinded fashion, on an intention-to-treat basis.

### Study monitoring

A data monitoring committee (DMC) will be responsible for an interim review of the study with regards to whether the study is appropriately powered and to determine any significant unforeseen adverse events, especially with respect to the effect of blood pressure cuff inflation in high-risk groups. This interim review will performed at a 6 to 12-month time-point and the minutes of all DMC meetings will be shared with the Research Ethics Committee (REC).

## Discussion

The ERICCIN trial is intended to investigate whether RIC, a simple non-invasive and low-cost intervention, can significantly reduce the rate of CIN in patients undergoing elective and semi-emergent coronary angiographic procedures, and whether improvement in biochemical markers are related to a persisting benefit in terms of renal function measured after 3 months of follow-up.

RIC has the potential to meet an unmet clinical need: current recommendations of using pre-hydration with *N*-acetyl cysteine and/or sodium bicarbonate and the use of iso-osmolar contrast media are presently best practice, and yet there remains a significant rate of contrast-induced nephropathy: Mehran and colleagues [8] report rates of between 7.5 and 57.3 % dependent upon the risk profile of the patients studied. Even where medical management is optimised, as was the case in the CARE [9] and REMEDIAL [28] studies, the overall rate of CIN remains high at around 10 %. RIC, however, has the prospect of significantly attenuating the rate of CIN. Three recent studies, using either arm-cuff RIC [21, 23] or intermittent cardiac RIC by intermittently inflating the catheter balloon in patients managed for non-ST elevation myocardial infarction [22], have shown promise in terms of attenuating the rate of CIN, reducing the rate of acute kidney injury by up to 60 %. Similarly favourable data have been seen using pharmacological conditioning, with iloprost administration reducing the rate of CIN by 68 % [29]. Therefore, conditioning appears to offer what cardiologists have been seeking: an effective intervention against CIN. The three existing RIC trials were small, particularly the two studies using balloon-cuff inflation as their conditioning signal [21, 23], and two had surprisingly high rates of CIN (40 % [21], 29.5 % [22], respectively), which most likely, is related to the risk profile of the patients recruited at the respective institutions. Therefore, there is a need for additional clinical trials that reflect all-comer, lower-risk patient populations that are typical of the general cardiology population, before moving to larger, multicentre, morbidity and mortality outcome trials. Moreover, data showing a persistent benefit on preserving renal function is lacking, and it is these gaps in our knowledge that the ERICCIN trial aims to bridge.

The biomarkers used in the present study are renal function, determined by eGFR/creatinine, serum NGAL and proteinuria, the latter determined both by the use of a simple and cheap urinary dip-stick and a urinary albumin:creatinine ratio. ERICCIN will be the first RIC trial to determine long-term (3 month) renal function following the initial early monitoring period between 48 and 72 h post-contrast exposure. This will enable us to determine the rate of persistent renal dysfunction as identified by Maioli et al. [4], and whether this rate of persistent renal dysfunction is ameliorated by RIC. NGAL is an emerging biomarker of acute kidney injury, with equivalent increases of both urinary and plasma NGAL within 6 h of renal the insult [30], a time course that, should it be reciprocated in angiographic patients, could influence hospital discharge decisions on patients admitted for day-case angiographic procedures. Proteinuria is, in itself, a risk factor for the onset of CIN [31]. Moreover, the onset and persistence of proteinuria is a recognised adverse prognostic factor [32–34] and its appearance following an angiographic intervention could be a predictor of later renal deterioration and cardiovascular mortality. Therefore, the detection of proteinuria has the potential to identify a high-risk sub-group within the CIN cohort. The ERICCIN trial will, therefore, provide biologically relevant data and provide practical tools for monitoring patients moving through coronary catheter laboratories.

In summary, ERICCIN is a single-centre, randomised double-blinded placebo-controlled trial that will investigate whether RIC can improve the rate of CIN following iodinated contrast exposure occurring following coronary angiographic procedures. It will follow patients for 3 months and will determine biochemical outcome data as well as collecting data related to cardiovascular and all-cause mortality for the duration of follow-up. The findings will have the potential to influence a change of clinical practice: RIC is a simple, non-invasive intervention that is cheap to deploy in an area where there are few, if any, effective alternatives.
